# The buckling instability of aggregating red blood cells

**DOI:** 10.1038/s41598-017-07634-6

**Published:** 2017-08-11

**Authors:** Daniel Flormann, Othmane Aouane, Lars Kaestner, Christian Ruloff, Chaouqi Misbah, Thomas Podgorski, Christian Wagner

**Affiliations:** 1 Experimental Physics, Saarland University, 66123 Saarbrücken, Germany; 2 Laboratoire Interdisciplinaire de Physique, UMR 5588 CNRS and Université Grenoble Alpes, B.P. 87, 38402 Saint-Martin-d’Hères Cedex, France; 3 Theoretical Medicine and Biosciences, Saarland University, Building 61.4, 66421 Homburg, Saar Germany; 4 Physics and Materials Science Research Unit, University of Luxembourg, 1511 Luxembourg, Luxembourg

## Abstract

Plasma proteins such as fibrinogen induce the aggregation of red blood cells (RBC) into rouleaux, which are responsible for the pronounced shear thinning behavior of blood, control the erythrocyte sedimentation rate (ESR) – a common hematological test – and are involved in many situations of physiological relevance such as structuration of blood in the microcirculation or clot formation in pathological situations. Confocal microscopy is used to characterize the shape of RBCs within rouleaux at equilibrium as a function of macromolecular concentration, revealing the diversity of contact zone morphology. Three different configurations that have only been partly predicted before are identified, namely parachute, male-female and sigmoid shapes, and quantitatively recovered by numerical simulations. A detailed experimental and theoretical analysis of clusters of two cells shows that the deformation increases nonlinearly with the interaction energy. Models indicate a forward bifurcation in which the contacting membrane undergoes a buckling instability from a flat to a deformed contact zone at a critical value of the interaction energy. These results are not only relevant for the understanding of the morphology and stability of RBC aggregates, but also for a whole class of interacting soft deformable objects such as vesicles, capsules or cells in tissues.

## Introduction

**Figure 1:**
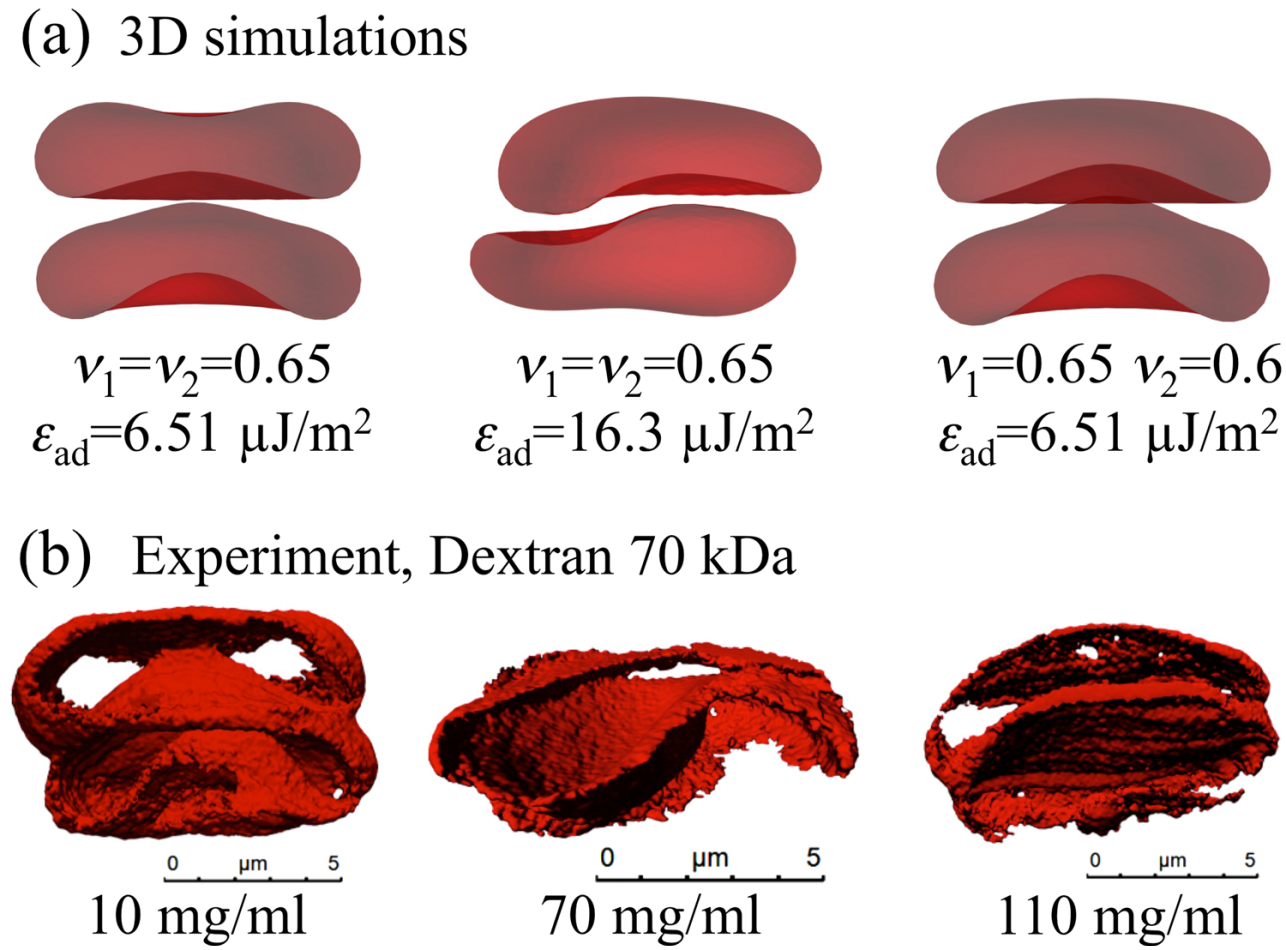
(**a**) Theoretical results (3D simulations) for RBC interaction and resulting contact zones for different reduced volumes *ν* and adhesion energies *ε*_*ad*_. (**b**) 3D-confocal imaging of RBC-doublets. The experimental results qualitatively reproduce the theoretical predictions.

Red blood cells (RBCs) have been a model system and an inspiration for the biophysics of the membrane for decades^1^. Among the numerous works in the literature, many studies have been devoted to adhesion of cells or vesicles and capsules on *flat and solid* surfaces^2, 3^, where, in the case of elastic capsules only, a buckling instability may occur due to the interplay between bending and stretching energy^4^. Buckling has also been observed in freestanding capsules due to osmotic pressure^5, 6^ or shear forces by external flow^7, 8^, and in vesicles due to asymmetric lipid distributions^9^ or shear forces by external flow^10^. The influence of intercellular adhesion^11^ is less known despite its relevance in dense environments such as blood at physiological hematocrit or even tissues. Theoretical studies on aggregation between two RBCs based on minimizing the free energy of the membrane^12, 13^ indicate a significant change of the geometry of contact zones between cells when varying the interaction energy. Some of these shapes have been observed experimentally^14^ but not quantified as a function of the dextran or fibrinogen concentrations. A variety of membrane shapes has been predicted theoretically for RBC doublets^13^, depending mainly on the nondimensionalized reduced adhesion strength *γ* and the reduced volume $\nu =3V\sqrt{4\pi }{S}^{-\mathrm{3/2}}$ where *V* and *S* are the enclosed volume and surface area of the membrane, a parameter that is indeed physiologically related to the hydration state and age of RBCs and can vary over a significant range. Examples include male-female (convex-concave) shapes and more or less pronounced sigmoid shapes obtained by varying *ν* and *γ*. Our 3D numerical simulations (Fig. 1(a)) show the same variety of contact zone shapes as well as the 3D reconstructions of our experimental confocal images (Fig. 1(b)). Experimental images also show that the amplitude of the deformation of contact zones depends non monotonically on macromolecular (dextran) concentration.

There are direct physiological implications of aggregation since RBCs in blood in stasis are known to form aggregates in the form of so called *rouleaux*. This clustering process is reversible and typical hydrodynamic shear forces in physiological flow are usually sufficient to break *rouleaux*, at least in larger vessels. In the 1960’s, fibrinogen could be identified as one of the main plasma proteins causing RBC aggregation or clustering^15–17^. It is known that the number and size of aggregates increase with fibrinogen concentration in the physiological range (from approximately 3 *mg*/*ml* for healthy adults up to 10 *mg*/*ml* in acute inflammatory phases). The level of RBC aggregation is used in one of the most fundamental standard hematological blood tests, the erythrocyte sedimentation rate (ESR) which is carried out worldwide many thousand times each day. The more and the larger the aggregates, the faster the sedimentation of the RBCs: by simply measuring visually the sedimentation front in a standardized glass capillary one gets a robust and quick, if unspecific, indication on the inflammatory state of the patient. Of course, the tendency of the RBCs to form aggregates may also increase the risk of thrombosis and cardiovascular diseases, especially in combination with stenosis.

The molecular mechanisms of macromolecular induced RBC aggregation have been the subject of controversial studies^18–23^. Two models have been proposed: One is based on the physical effect of depletion^24–28^ and the other on physisorption or bridging^15, 29–32^. In addition to the specific adhesion mechanism, the strength of RBC aggregation also depends on their physical properties, such as deformability^33^, surface charge^34^ and reduced volume^12, 13, 35^. It can also be induced by other macromolecules such as dextran, a widely used molecule in laboratory experiments or as a plasma expander and in veterinary medicine. AFM based single cell force spectroscopy, rheology and sedimentation of RBCs^28, 36, 37^ as well as theoretical models^23^ depict that an increasing dextran concentration leads first to an increase of the interaction strength among RBCs up to a specified macromolecule concentration. Beyond that maximum the interaction strength decreases again, and cell–cell aggregation vanishes. This leads to a characteristic bell–shaped adhesion-energy versus concentration curve. For the case of fibrinogen, it was found the interaction energy increases with the concentration, but data from the literature are typically limited to lower concentrations than for the case of dextran^28, 36–38^.

We present here a systematic experimental study in order to analyze quantitatively the evolution of the contact zone as a function of aggregating molecule concentration, in a large domain of parameter space by using either dextran or fibrinogen. We analyze the case of RBC doublets as well as that of larger *rouleaux*. A systematic numerical investigation supports the experimental results. The numerical results show that in the case of two adhering cells, the interface remains flat up to a critical value of interaction energy above which it bifurcates into a deformed, buckled state. Our analytical model reveals a forward bifurcation with critical parameters close to those of the full numerical problem.

## Analysis of RBC doublet shapes

### Experiment

**Figure 2:**
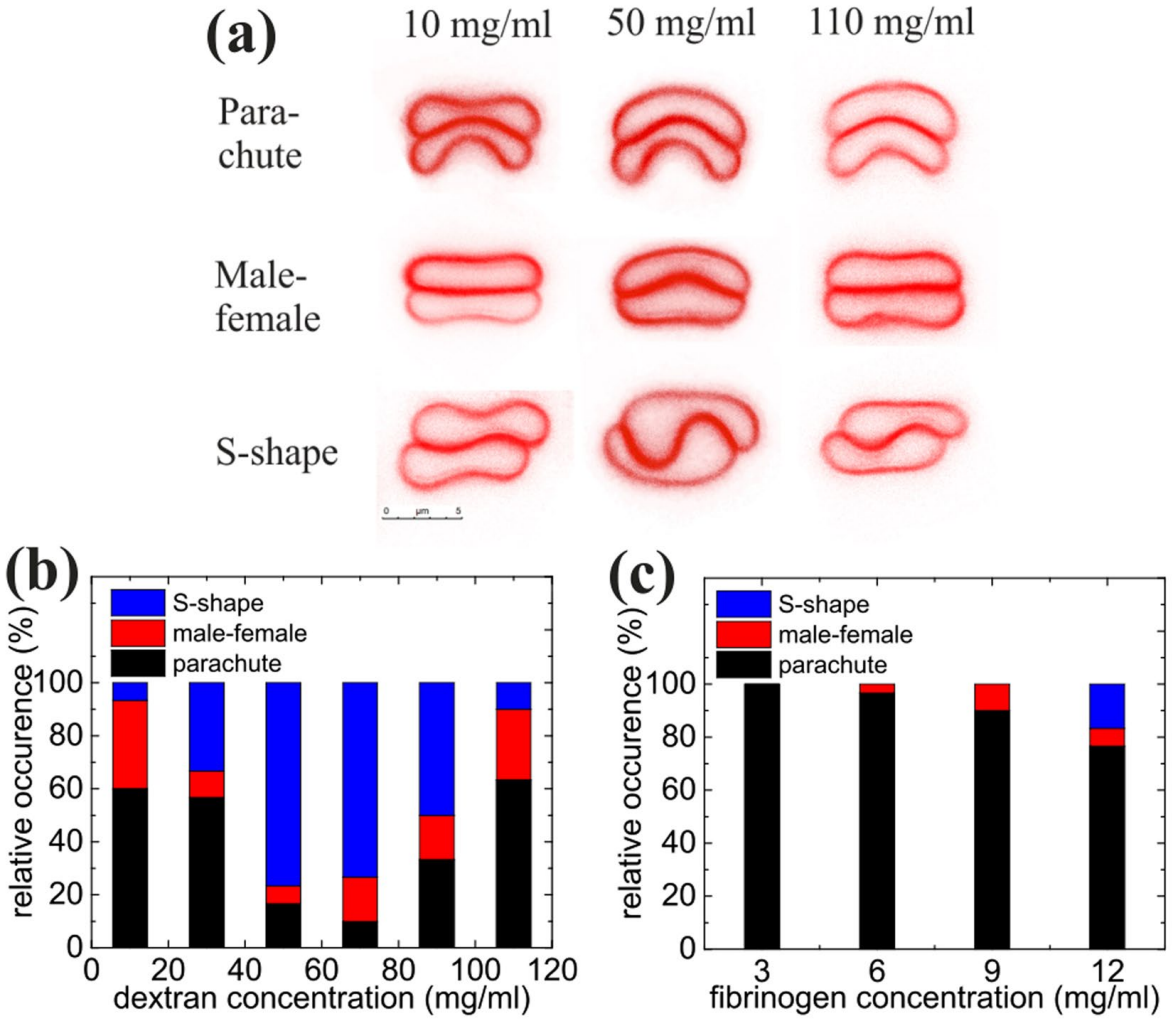
(**a**) Representative shapes of sedimenting doublets for different concentrations of dextran. At each concentration three different types of shapes can be identified: parachute, S-shape and male-female shape. (**b**) Distribution of male-female, parachute and S-shapes for different concentrations of sedimenting cells in discocyte shape in dextran solutions. (**c**) Distribution of male-female, parachute and S-shapes for different concentrations of sedimenting cells in discocyte shape in fibrinogen solutions.

The analysis of the interfacial shape of two cells *rouleaux*, here called doublets, was performed by confocal imaging of doublets during free sedimentation, i.e. while not in contact with surfaces. Typical images are shown in Fig. 2 for doublets in dextran 70 kDa solutions (see S.I. for the fibrinogen case). There is a significant evolution of the interfacial shape with polymer concentration: At low and high concentrations the interface is only weakly deformed while in the intermediate range where interaction energy is higher, the interface is strongly bent. Qualitatively, three different kinds of interfacial shapes were identified: parachute, male-female and sigmoid. In the sigmoid (S-shape interface), cells were mostly laterally displaced, with a non-monotonous curvature of the contact zone, while in other shapes the two cells had a common symmetry axis. The parachute shape was defined by at least one of the cells having a convex and a concave side. The male-female shape was defined when the interface had a bulge, with rather flat non-interacting large membrane parts. Cells with a flat interface were also classified under this category. Similar shapes were found for fibrinogen solutions. As can be seen in Fig. 2(a), all shapes can be found for all polymer concentrations, probably due to dispersity of cell properties in the sample, with a probability that depends on concentration.

A quantitative analysis of the distribution of the different interfacial shapes for the different concentrations of dextran is shown in Fig. 2(b), revealing an increase of the occurrence of sigmoid shapes in the intermediate range of polymer concentrations, i.e. at larger interaction energy favoring slightly larger contact areas. At low and high concentrations of dextran, doublets are mostly in the parachute (about 60%) and male-female shapes. For fibrinogen (Fig. 2(c)), mostly parachute shapes were observed in the physiological range of the protein’s concentration. The case of echinocyte RBC doublets, obtained after sedimentation and contact with glass, was also studied (see S.I.).

**Figure 3:**
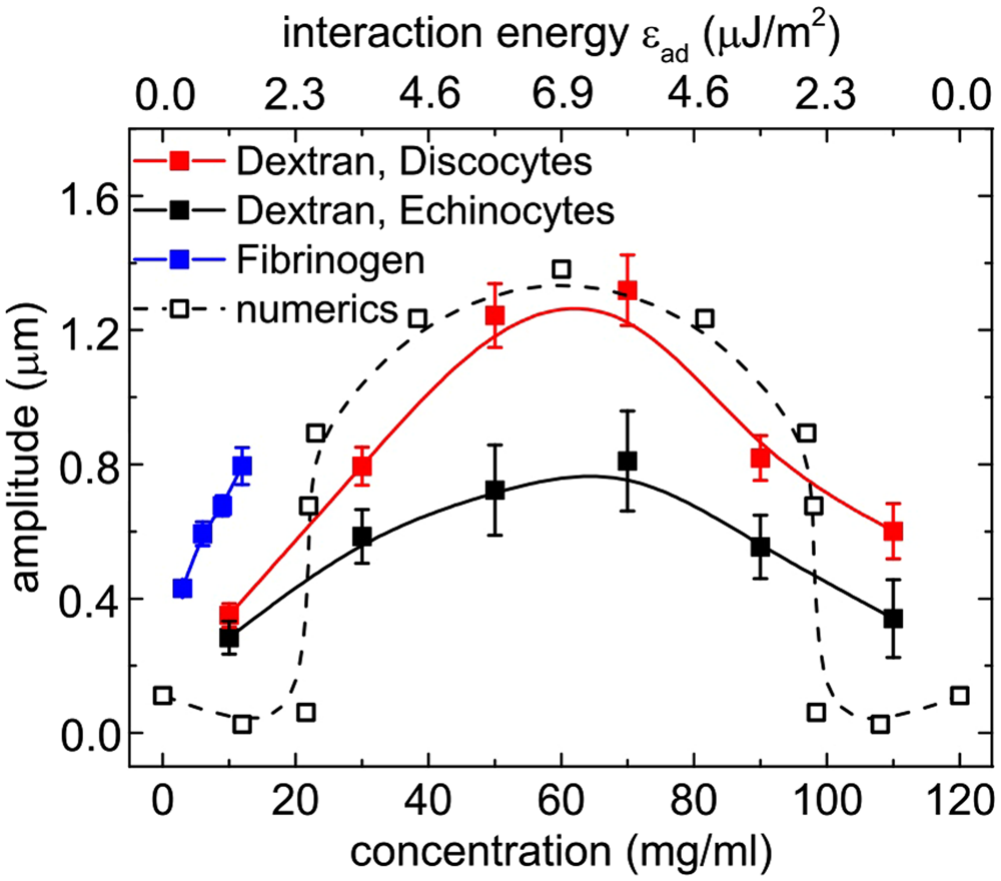
Amplitude of the interfacial deformation for varying concentrations of dextran 70 kDa for discocyte and echinocyte membrane shapes in the experiments and a reduced area of *τ* = 0.65 in the numerics. For numerical results, an energy scale was chosen by assuming a piece-wise linear variation of the interaction energy with concentration with a maximum of 22.5 *μ*J/m^2^ at 60 mg/ml. Error bars show standard errors.

Quantification of the interfacial shape deformation was done by fitting it with a heuristically chosen sinus function (see S.I.), whose amplitude was taken as a measure of the interfacial curvature (Fig. S2). A bell shape dependency of the deformation amplitude on polymer concentration is seen (Fig. 3), similar to the dependency found in AFM single cell force spectroscopy^28^ or aggregation index (see S.I.). For the case of dextran, both doublets with discocytic and with echinocytic cells were analysed. The amplitude for discocytic cells can reach much larger values than echinocytes that have a close-to-spherical shape and probably a higher membrane stiffness^35^.

### Simulation and theory

In order to gain further insight on the variability of shapes and estimate the interaction energies corresponding to the experimental doublets and we performed dynamic numerical simulations in which two model RBCs with a membrane interaction potential are placed next to each other in a fluid and left to evolve until steady state is reached (see S.I. for details of the method). Based on a set of 3D simulations provided below for comparison and several previous successful studies (especially equilibrium shapes and shape dynamics under external flow) which have shown that 2D and 3D models capture the same essential features^39–41^, we focus here on a quantitative analysis based on a 2D model. In 2D the notion of in-plane shear elasticity looses its meaning and the vesicles and inextensible capsule model (often evoked to model RBC) are equivalent.

**Figure 4:**
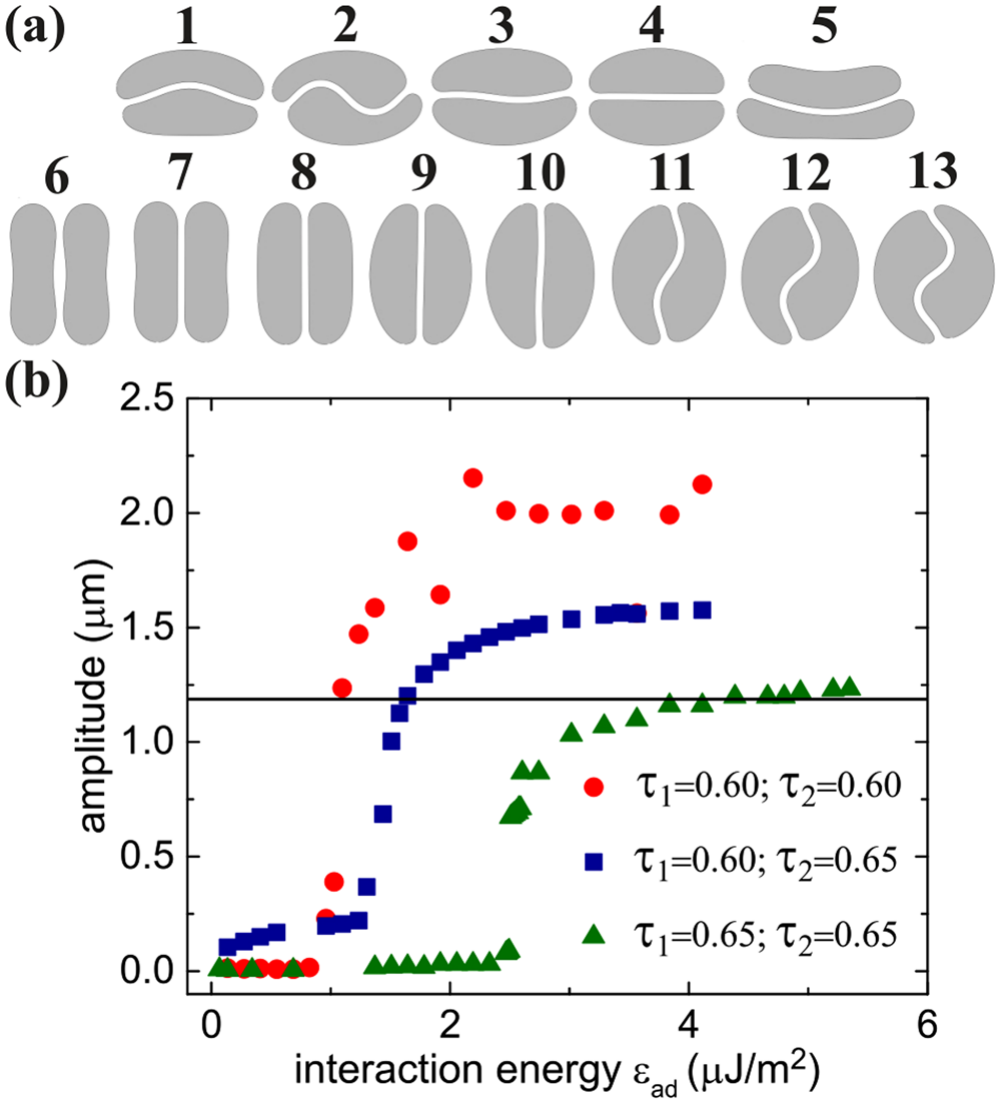
(**a**) 2D simulation of vesicles doublets. (1–5) for the same interaction energy *ε*_*ad*_ = 1.16 *μ*J/m^2^ and using different combinations of reduced areas: (1) *τ*_1_ = 0.50 and *τ*_2_ = 0.65; (2) *τ*_1_ = *τ*_2_ = 0.60; (3) *τ*_1_ = 0.60 and *τ*_2_ = 0.65; and (4) *τ*_1_ = *τ*_2_ = 0.65, one identifies male-female shape (1), S-shape (2,3), and flat contact interface (4). (5) a parachute shape aggregate observed when considering the membrane stiffness as a variable; the used parameters read as *τ*_1_ = 0.55 and *τ*_2_ = 0.40, *κ*_*B*1_ = *κ*_*B*2_ = *κ*_*B*_/4, and *ε*_*ad*_ = 0.025 *μ*J/m^2^. (6–13): 2D simulation of doublets for different interaction energies: from (6) to (13) *ε*_*ad*_ = 0, 0.07, 0.34, 1.37, 2.47, 2.54, 3.02, 5.21, 8.23 *μ*J/m^2^. The reduced area of both cells is fixed to *τ*_1_ = *τ*_2_ = 0.65. (**b**) The amplitude of deformation of the contact zone between the doublets as function of the interaction energy for different combinations of reduced areas.

**Figure 5:**
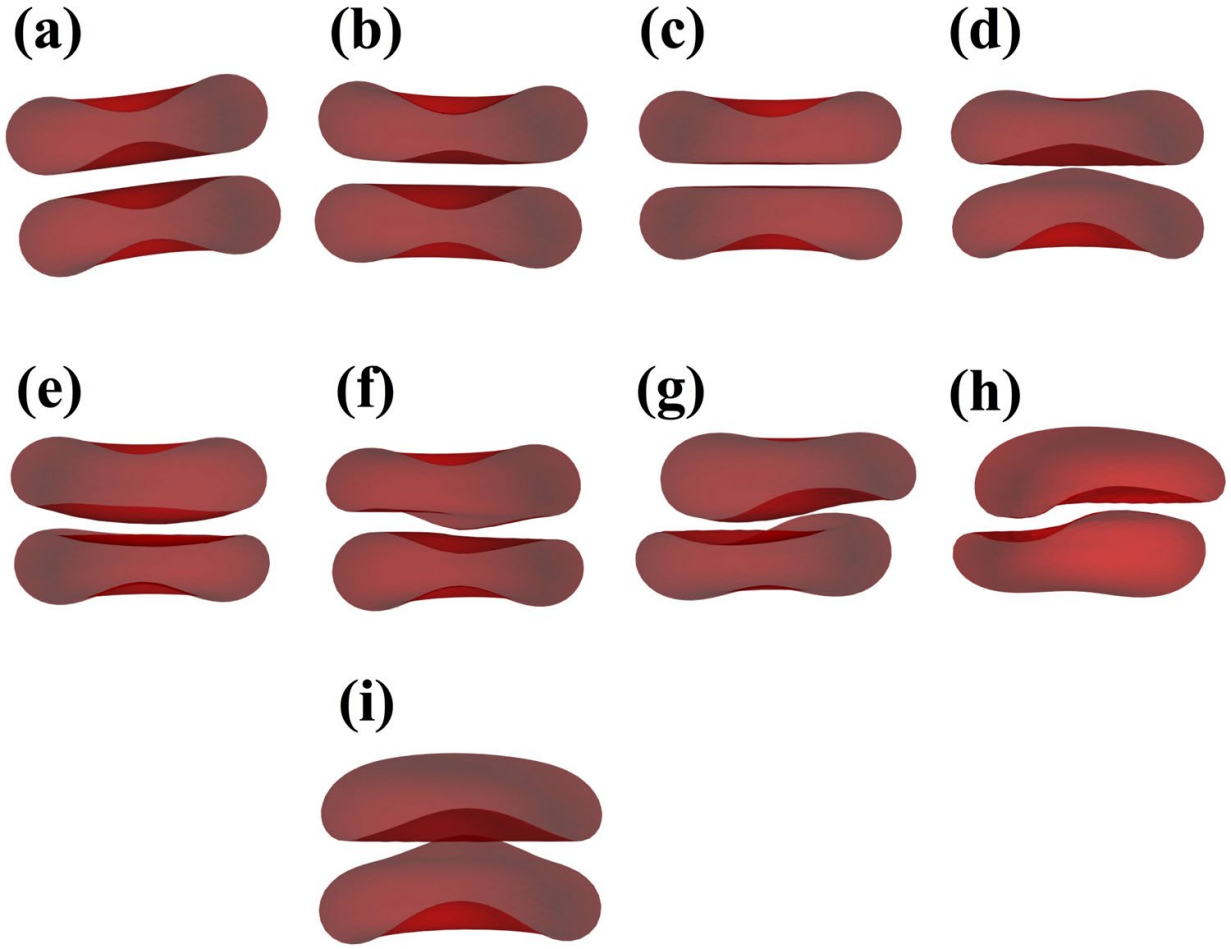
3D simulations of interacting RBC doublets. (**a**–**h**) Identical RBCs (*ν*_1_ = *ν*_2_ = 0.65) for an increasing interaction energy (in *μ*J/m^2^: (**a**) *ε*_*ad*_ = 0.98, (**b**) *ε*_*ad*_ = 1.95, (**c**) *ε*_*ad*_ = 3.24, (**d**) *ε*_*ad*_ = 3.90, (**e**) *ε*_*ad*_ = 4.88, (**f**) *ε*_*ad*_ = 6.51, (**g**) *ε*_*ad*_ = 9.76, (**h**) *ε*_*ad*_ = 16.3), showing a transition towards sigmoid shapes between the (**e**,**f**) cases. (**i**) Asymmetric case with *ν*_1_ = 0.65 and *ν*_2_ = 0.6 and *ε*_*ad*_ = 6.51 *μ*J/m^2^ showing a parachute shape.

For reduced areas *τ* = 4*πS*/*L* ^2^ (where *S* is the area of the 2D vesicle and *L* its perimeter) in the range (0.40,0.65) with different combinations (cells with same or different reduced areas) and a membrane stiffness of *κ*_*B*_ = 4 × 10^−19^ J, we varied the interaction energy *ε*_*ad*_ in the range 0–8.23 *μ*J/m^2^. The resulting interfacial shapes for different combinations of reduced area *τ*, interaction energy *ε*_*ad*_ and curvature energy *κ* (Fig. 4) were in qualitative agreement with the experimentally obtained shapes, namely male-female, S-shapes and parachute shapes, as well as with a set of 3D simulations (Fig. 5).

Figure 4(a) shows the evolution of the deformation as a function of the interaction energy. As for the analysis of experimental data we fit the interfacial shape with a sine function. The mean amplitude of the fitted curve was compared to experimental results (Fig. 3), by heuristically choosing a correspondence between concentration and interaction energy: We assume a linear increase of the interaction energy with concentration up to 6.9 *μ*J/m^2^ at 60 mg/ml, followed by a symmetric linear decrease, in order to get a good compromise between an agreement on the width and the maximum value of bell shaped curves. This piecewise linear variation of the interaction energy is a simplification which nevertheless yields a qualitative agreement for the overall behavior. Interestingly, the numerical results also show that the transition from a flat to a deformed interface only occurs at some finite interaction energy *ε*_*ad*_ (Fig. 4(b)). In principle, this method could allow the determination of the experimental interaction energy for a given concentration. However, the numerical results depend quite sensitively on the reduced areas *τ* of the two cells (Fig. 4(b)), a parameter that varies slightly between cells in experiments, even in the same sample. This dispersity is probably also the reason why the bell shape in Fig. 3(a) of the experimental data does not show this sharp transition.

**Figure 6:**
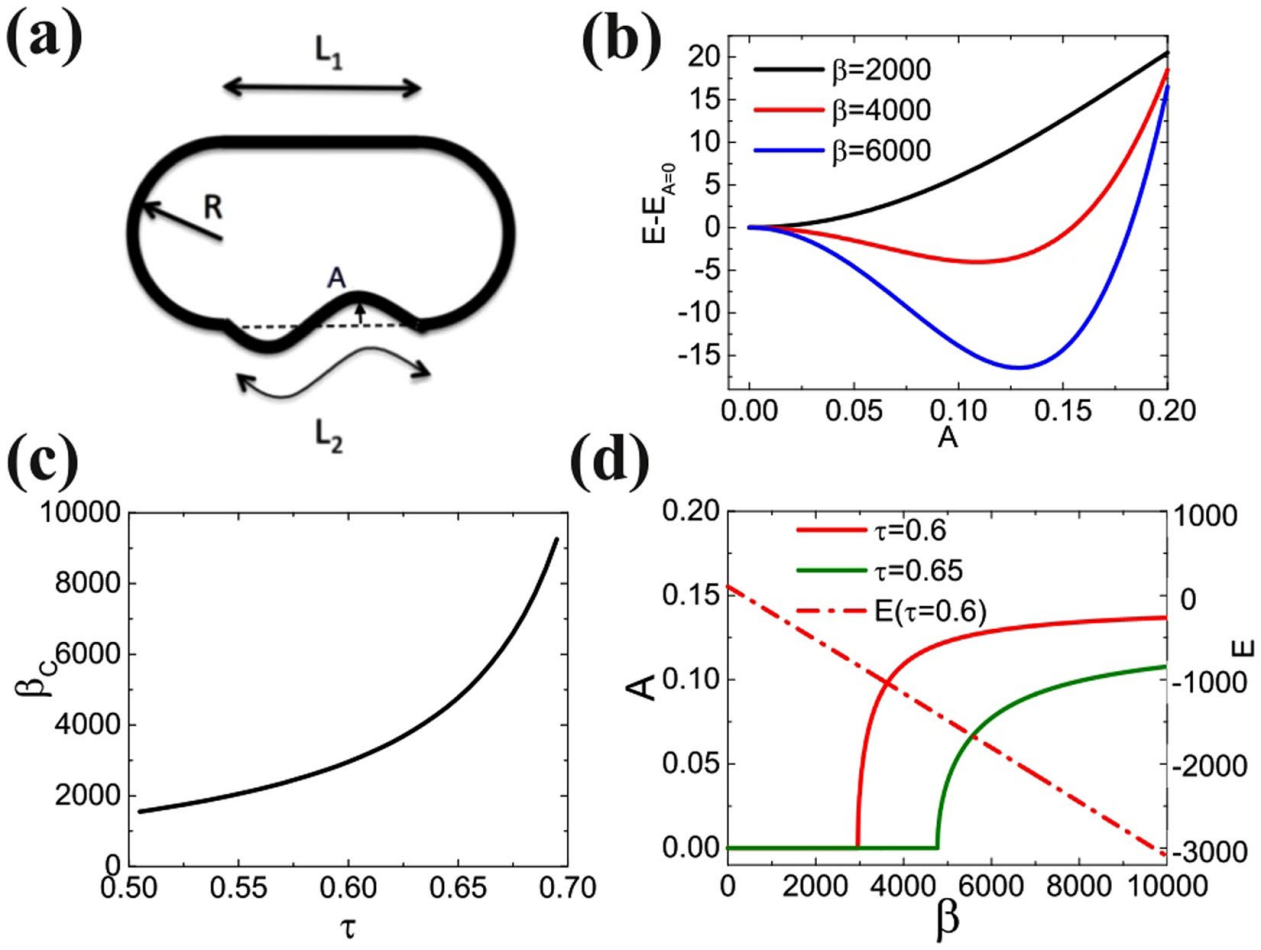
Analytical model: (**a**) Notations; (**b**) Total energy vs. amplitude for increasing adhesion energy; (**c**) buckling threshold vs. reduced area; (**d**) bifurcation diagram for *τ* = 0.6 and 0.65 and variation of the energy.

The evolution of the amplitude of contact zone’s deformation in Fig. 4(b) suggests that a bifurcation akin to buckling occurs at a critical value of the interaction energy, triggering a change of shape from flat to S-shape. To get some insight into the involved mechanism, this behavior can actually be recovered by an analytical model through a minimization of the energy of the system. Let us suppose that the shape of an RBC in a symmetric doublet (in 2D) is approximated as shown in Fig. 6(a), with two semi-circular caps of radius *R*, a roughly straight portion where curvature is negligible (length *L*_1_) and a contact zone with a sinusoidal shape of curvilinear length *L*_2_ and amplitude *A* described by the equation:

1 $$y(x)=A{L}_{1}\,\sin \,\frac{2\pi x}{{L}_{1}}$$

for −*L*_1_/2 < *x* < *L*_1_/2. The corresponding interaction energy (per unit length in the third dimension) is, at order 4 in *A*:

2 $${E}_{i}=-{\varepsilon }_{ad}{L}_{1}\,(1+{A}^{2}{\pi }^{2}-\frac{3}{4}{\pi }^{4}{A}^{4}).$$

where *ε*_*ad*_ is the interaction energy per unit area.

The curvature energy of the system is concentrated in the circular caps *E*_*b*1_ and the deformed contact zone *E*_*b*2_ given by:

3 $${E}_{b1}=\frac{2\pi \kappa }{R}$$

4 $${E}_{b2}\simeq \kappa \frac{8{\pi }^{4}{A}^{2}-20{\pi }^{6}{A}^{4}}{{L}_{1}}$$

The total energy *E* = *E*_*i*_ + *E*_*b*1_ + *E*_*b*2_ therefore depends on *A*, *R* and *L*_1_ which are related through the constant area *S* and constant perimeter *L* constraints.

In the following we define a dimensionless energy E* = *EL*/*κ*, a rescaled interaction energy *β* = *ε*_*ad*_ *L* ^2^/*κ*, and rescale all lengths by *L*: R* = *R*/*L* and so on, and introduce the reduced area *τ* = 4*πS*/*L* ^2^. The total energy E* can then be derived and expanded at order 4 in *A* (see supplemental material). Figure 6(b) shows the energy due to deformation for *τ* = 0.6 and different interaction energies *β*. Below a critical value *β*_*c*_, the minimum of energy corresponds to *A* = 0 (flat interface), while for *β* > *β*_*c*_, the energy is minimal for a finite value of *A* = *A*_*eq*_ (see S.I.).

This defines the bifurcation threshold *β*_*c*_ corresponding to *A*_*eq*_ = 0, which is an increasing function of *τ* (Fig. 6(c)):

5 $${\beta }_{c}=\frac{8{\pi }^{2}\,(9\sqrt{1-\tau }-8)}{-2\sqrt{1-\tau }\tau +3\tau +3\sqrt{1-\tau }-3}$$

The bifurcation diagram of the deformation amplitude (Fig. 6(d)) reveals a classical supercritical bifurcation, owing to the competition between interaction energy and curvature energy through the constant perimeter and area constraints. Orders of magnitude of the bifurcation threshold and amplitude of the deformation are in good agreement with experiments and numerical simulations. We have *ε*_*ad*_ = *κβ*/*L* ^2^. If we take *L* ~ 24 *μ*m and *κ* ~ 4 × 10^−19^ J as in simulations, the critical interaction energy is *ε*_*c*_ ~ 2 *μ*J/m^2^ for *τ* = 0.6 and *ε*_*c*_ ~ 3.2 *μ*J/m^2^ for *τ* = 0.65, which is in reasonable agreement with the simulations of Fig. 4(b), while the amplitude of the deformation *A* saturates at ~0.13 *L* = 3.1 *μ*m in this model, rather close to the value ~2 *μ*m found in the simulations given the simplicity of the model.

Interestingly, the energy of the equilibrium configuration decreases linearly with *β* as shown in Fig. 6(d), with no sign of the shape bifurcation due to the smallness of the non linear terms arising from the deformation in the curvature and interaction energy terms. This result can be of interest in the modeling of clustering dynamics in flow for instance, where the dissociation rate due to hydrodynamic stresses can be assumed to simply scale like 1/*ε*_*ad*_ despite the possibly complex shapes of contact zones.

## Analysis of membrane shapes in larger RBC clusters

**Figure 7:**
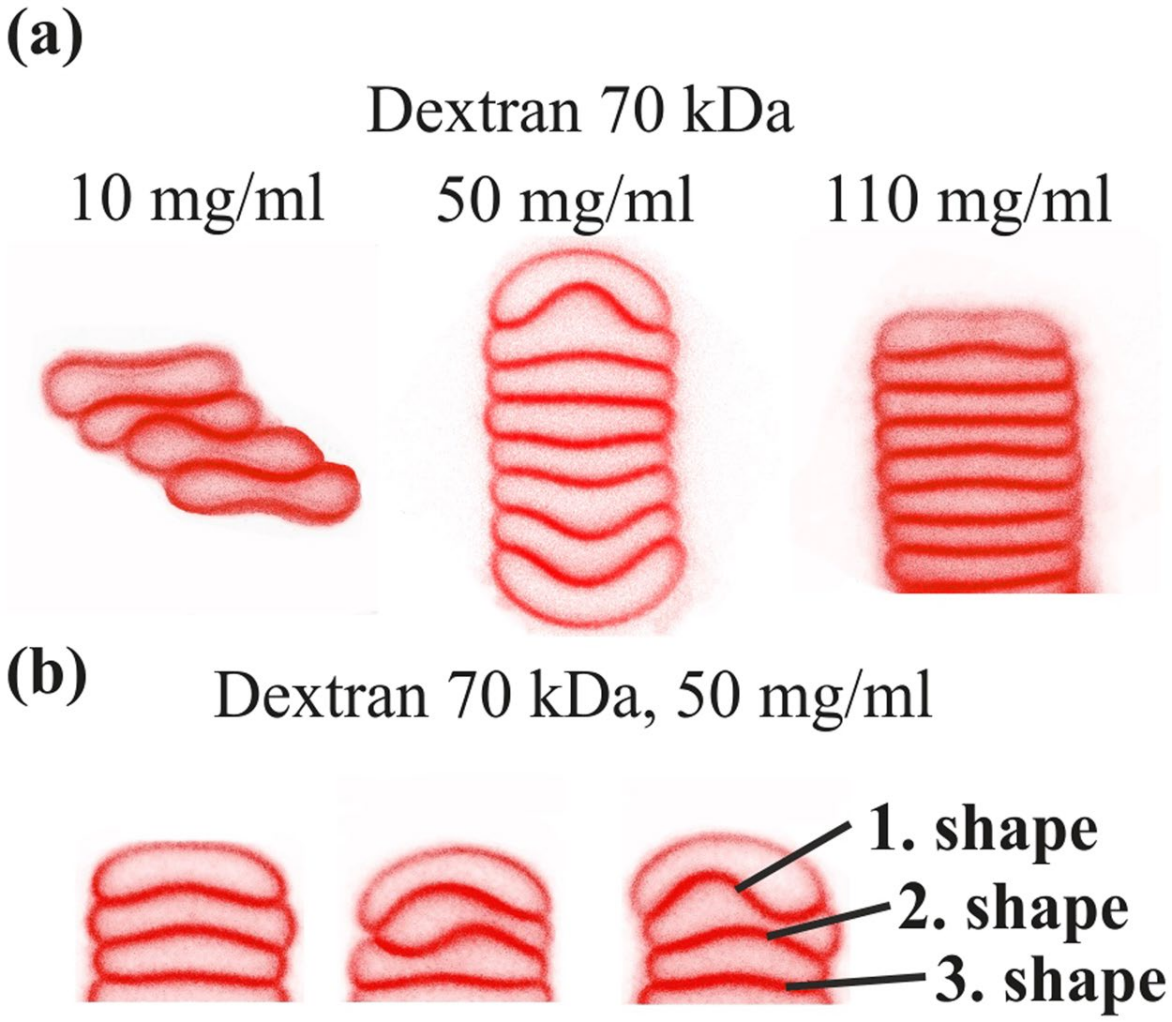
(**a**) Representative images of larger clusters (rouleaux) at different concentrations of dextran 70 kDa. (**b**) The first three interfaces show a large variation in shapes even at constant polymer concentration and we observe, male-female and S-shapes.

A characterization of larger clusters was performed with *rouleaux* of at least 7 cells. The fast sedimentation and arbitrary orientation of the rouleaux only allowed us to image them when settled on the (BSA treated) cover slip. Since fibrinogen led to significant inter-connections between *rouleaux* which eventually led to a percolated gel like structure at 45% hematocrit, we only focused on rouleaux in linear configuration in dextran solutions (Fig. 7a). At 10 mg/ml the cells in a *rouleau* were typically laterally shifted and we did not observe large clusters. At higher concentrations of dextran larger *rouleaux* were observed and as shown in Fig. 7, only the first three interfaces exhibited significant deformation (Fig. 7a) while the interfaces far from the tip were always in a male-female shape with very low deformation, mostly below the experimental resolution limit. Again, a large variety of shapes was observed for these first three interfaces (Fig. 7b).

**Figure 8:**
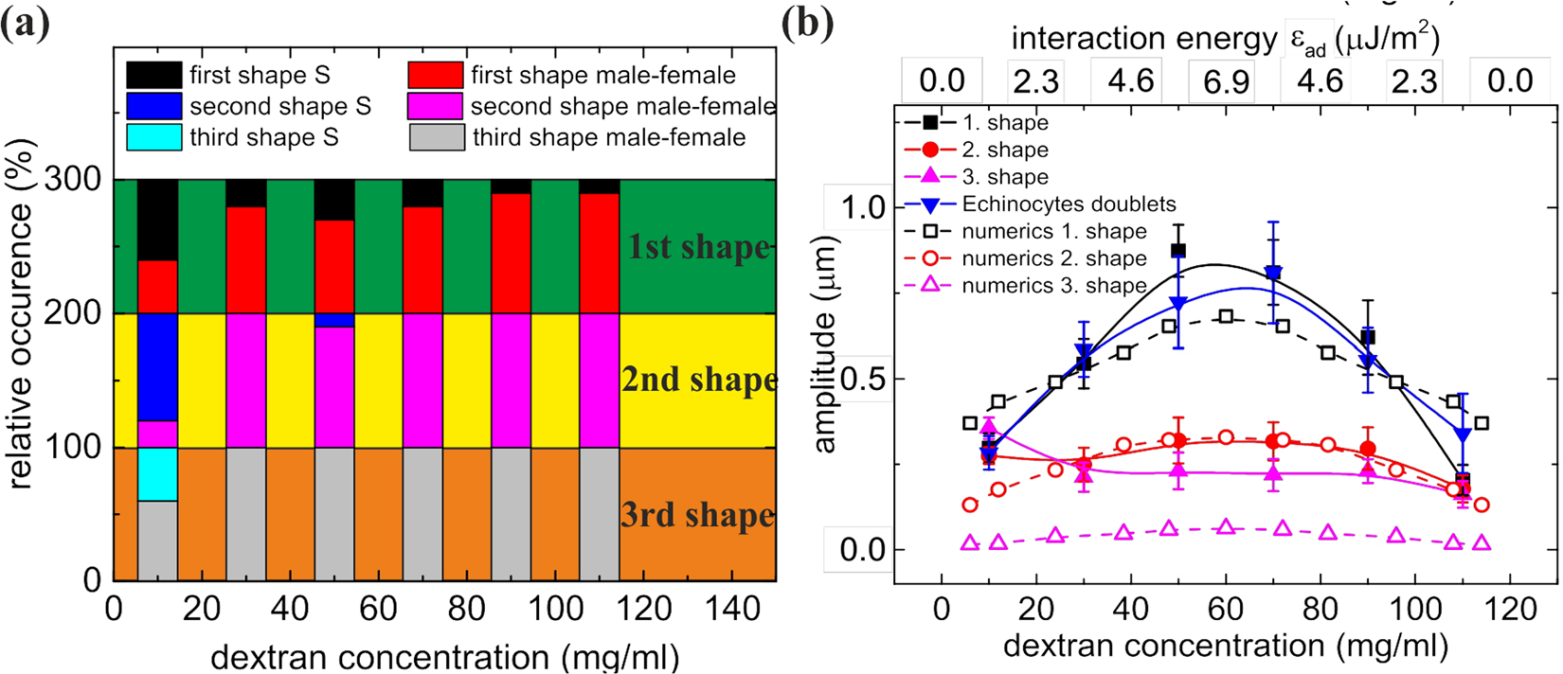
(**a**) Distribution of shapes within rouleaux for the first three contact zones. Only male-female and S-shapes were observed. (**b**) Amplitudes of deformation of the first three interacting membranes vs. dextran concentration in experiments and vs. interaction energy for numerics. For comparison the amplitudes of the shapes of the echinocytic doublets are shown as well.

As for doublets, these interfaces can be sorted into different categories and the distribution of shapes is shown separately for the first, second and third interface starting from the doublet tip in Fig. 8a. Unlike the case of doublets, we observed mostly male-female shapes and no parachute shapes at all, likely due to the constraints induced by the next attaching cell. For concentrations in the lower to medium-range, S-shape contact zones exist more frequently for the first contact zone than for the next ones, while male-female shapes are indeed prevalent everywhere except at very low dextran concentrations where RBCs tend to stay very close to their free discoid shape due to the low interaction energy, in agreement with the theoretical results on low-aggregation behavior mentioned in ref. 12.

The amplitudes of the interfacial deformations (determined by the same method as for doublets) are shown in Fig. 8a. Again, a bell shape behavior for all three interfaces was observed. Interestingly, the amplitude of the first contact zone was very similar to the values measured for echinocyte doublets (see S.I.), too. Indeed, the aggregation into larger clusters gives less degree of freedom to RBCs due to higher constraints on the membrane, leading to a higher effective stiffness of RBCs.

**Figure 9:**
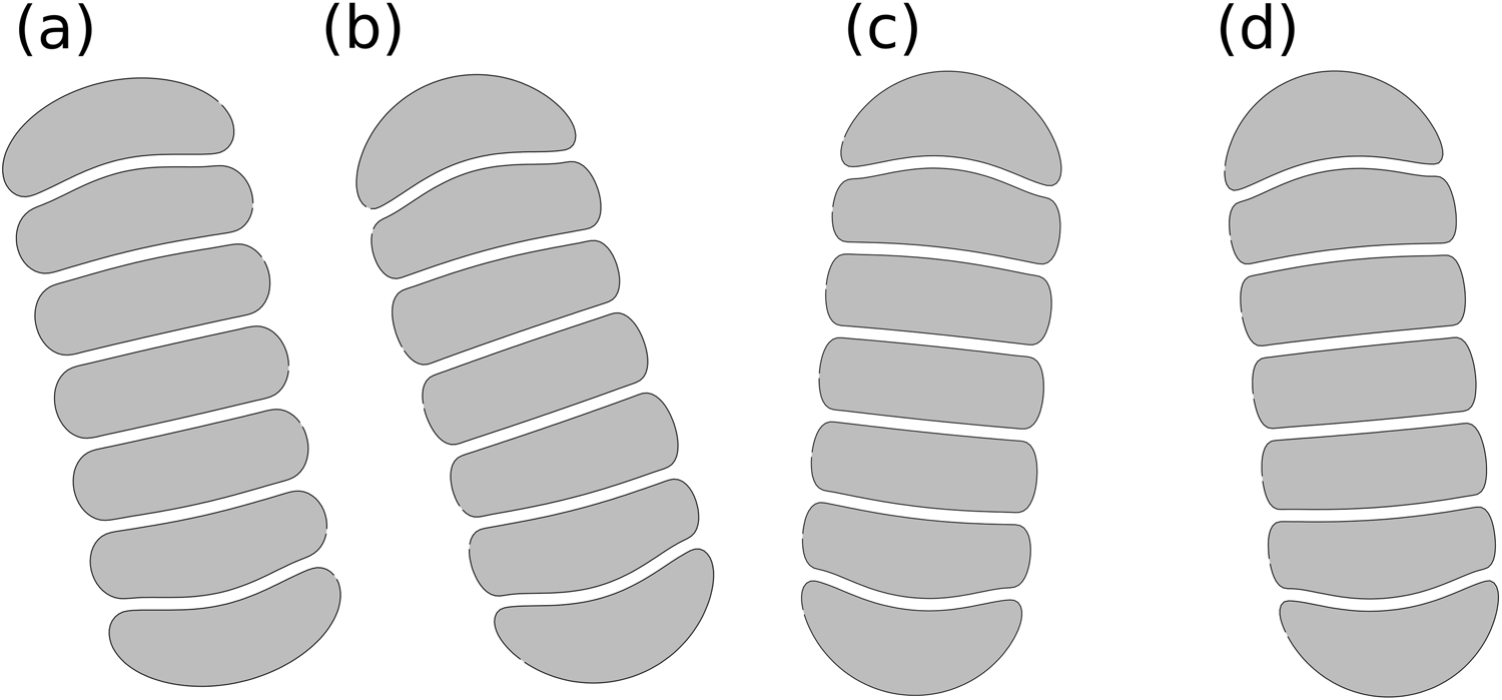
Simulations of clusters of 7 cells﻿: (**a**) *ε*_ad﻿_ = 0.67 µJ/m﻿^2^; (**b**) *ε*_ad_ = 2.55 µJ/m^2^; (**c**) *ε*_ad_ = 4.43 µJ/m^2^; (**d**) *ε*_ad_ = 6.93 µJ/m^2﻿^.

Numerical simulations were performed for 7 cells and different interaction energies *ε*_*ad*_, a constant reduced area *τ* = 0.65 and membrane stiffness *κ*_*B*_ = 4 × 10^−19^ J (Fig. 9). The results agree qualitatively with the experimental results and the amplitudes of the interfacial deformations of the first and second interface were in good agreement with the experimental data using the same energy scale as for doublets (see Fig. 8). However, the amplitudes between experiment and theory differed significantly for the third shape. Here the variability of the experimentally observed shapes plays a role, as well as the blurring of the contact zone due to resolution in experiments, which tends to increase the amplitude of the fitted sine function.

## Discussion

Red blood cells aggregate due to the presence of plasma proteins or biomimicking polymers. This is an example of adhesion between two very soft interfaces, different from the case of adhesion on a solid substrate, where we observe a buckling of the cell–cell interface. The nature of this instability is revealed by our numerical simulations and explained by an analytical model. The critical threshold of buckling and the final geometry of the aggregate depends strongly on the physical parameters, i.e. on the reduced volume of the cells. For large reduced volumes that correspond to more spherical cells, the critical interaction energy for buckling increases. In the case of doublets of two cells having identical reduced volumes close to that of a typical RBC, a sigmoid deformation of the interface takes place, while for two different reduced volumes male-female shapes can be obtained due to the asymmetry of properties that forces a different mode. We also characterized long linear clusters experimentally and numerically and have found that only the three interfaces at the two ends are considerably deformed. It would undoubtedly be interesting to check how these contact shapes obtained in quasi-static conditions evolve in pathological conditions, or under flow with the additional constraint of hydrodynamic stresses.

## Methods

Human blood withdrawal from healthy donors as well as blood preparation and manipulation were performed according to regulations and protocols that were approved by the ethic commission of the Ärztekammer des Saarlandes (reference No 24/12). We obtained informed consent from the donors after the nature and possible consequences of the studies were explained.

### RBC preparation

Venous blood of three healthy donors (one female, 26 years, blood group 0 negative; one male, 43 years, blood group 0 negative; one male, 27 years, blood group B positive) was drawn into conventional EDTA tubes (S-Monovette; Sarstedt, Nümbrecht, Germany) and all measurements were performed within 4 hours after the blood had been drawn. After washing the RBCs three times (704 g, 3 min) with phosphate-buffered solution (PBS, Life Technologies, Waltham, MA, USA) 0.1 *μ*l CellMask (Life Technologies, Waltham, MA, USA) was added for fluorescence labeling to 200 *μ*l RBCs in 1 ml PBS for 10 min. After washing two times (704 g, 3 min) the supernatant was removed and the RBCs were resuspended in solutions of dextran (Dextran 70 from Leuconostoc mesenteroides, Sigma-Aldrich, St Louis, USA) or (washed) fibrinogen (fibrinogen from human plasma, Sigma-Aldrich, St Louis, USA) in PBS. The fibrinogen as delivered contains approximately 60% Fibrinogen, 25% sodium chloride and 15% of sodium citrate. In order to remove the salt the solutions were ultrafiltrated with a 10 kDa filter (Vivaspin Turbo 4, Satorius AG, Goettingen, Germany) at 3500 g in a centrifuge (Hermle Z326K, Hermle Labortechnik GmbH, Wehingen, Germany) for 10 hours by adding 2 ml of PBS every hour. The remaining protein solution was freeze-dried and the proteins were resuspended in PBS. Fibrinogen was used in the measurements within 24 hours after freeze-drying and the osmolality of this solutions was verified to be in the order of PBS (+/−5%).

### Well preparation

Cylindrical wells of diameter 5 mm, height 1.6 mm (*μ*-Slide 18 Well-Flat from Ibidi, Munich, Germany) were used as sample chambers. RBCs sitting on the bottom slide of the well changed to echinocytes due to the glass effect. For the measurements in which we wanted to preserve the discoyte shape of sedimented cells, the wells were covered with 1 mg/ml bovine serum albumine (BSA, Sigma-Aldrich, St Louis, USA) in PBS for 30 min at 37 °C. The BSA-PBS solution was removed and after a drying time of 10 min at 37 °C the RBC suspensions were added.

### Imaging of the interfacial shape

All observations were performed with the confocal microscope described in the previous section at a temperature of 23 °C with an hematocrit of 0.2% for the imaging of RBC doublets and 0.4% for clusters larger than 6 RBCs. Images of doublets of RBC in their normal discoid shape could be recorded in free volume during the slow sedimentation process which prevented any contact with the glass surface of the cover slip. This procedure did allow to study the aggregates without any other influence but we should also note that it was also not possible to investigate the interfacial shape of sedimented cells in discocytic state. In this configuration, they were horizontally oriented with their interface and the RBC membrane was very thin. The optical 2-D sections showed a good signal only when the membrane was oriented along the axis of the point spread function of the laser focus. However, images of sedimented RBCs were also taken on the non-treated cover slips, i.e. for the case of echinocytes which were often oriented with their interface perpendicular to the cover slip, most likely due to their rounder shape. In total, 30 cells for discocytes and 15 for echinocytes were evaluated. For clusters that were larger than 6 cells, measurements could be only be performed on sedimented cells. In this case the cover slip was always treated with BSA to preserve the discocytic shape, and contact zones were always oriented perpendicular to the cover slip, thereby allowing a good characterization. The raw images were contrast enhanced and the position of the interfacial membrane contours was manually determined by visual inspection. The point cloud that indicated the interface was extracted with Matlab (The MathWorks GmbH, Ismaning, Germany) and stored into a one dimensional array to allow for a fitting procedure with Origin (OriginLab Corporation, Northampton, MA, USA). The interface was fitted with a heuristically chosen sinus function which yielded reasonable agreement. For sigmoid shapes the wave length of the sinus was left as a free parameter and for the male-female and parachute shape the wave length was limited to a range of ±20% around the double of the cell diameter.

### 3-D imaging

This accounts only for the images shown in Fig. 1. In the preparation step for the wells CellTak (CellTak, BC Bioscience, San Jose, USA) was added as specified by the manufacturer in order to immobilize the cells on the BSA treated cover slip. A stack of 50 images was taken at a distance of 0.1 *μ*m in z-direction, while the x-y area was 12 × 12 *μ*m. A surface rendering with Imaris (Bitplane AG, Zuerich, Switzerland) allowed to look through the cell membranes attached to the bottom onto the contact zones.

## Electronic supplementary material

Supplementary Information
