# Supplementary material for: The buckling instability of aggregating red blood cells
Source: Sci Rep. 2017 Aug 11;7:7928. doi: 10.1038/s41598-017-07634-6 (PMC5554189; doi:10.1038/s41598-017-07634-6)
Supplement: Supplementary file 1 — Supplementary Information [file 41598_2017_7634_MOESM1_ESM.pdf]

Supplementary Information - The buckling instability of aggregating red blood cells

Daniel Flormann, Othmane Aouane, Lars Kaestner, Christian Ruloff, Chaouqi Misbah, Thomas Podgorski, and Christian Wagner

Microscopic aggregation index

A simple way to quantitatively characterize the clustering of the RBCs is based on the quantification of sedimented aggregates within a certain area on a microscope slide. Chien and Jan[1] defined a microscopic aggregation index (MAI) as the ratio of the total number of cells to the total number of "particles" (individual cells and aggregates), representing the average number of cells per particle. Here, we investigate RBC aggregation by confocal microscopy and define the MAI as the the ratio of the number of aggregated cells at the bottom of the well after sedimentation to the total number of cells, i.e. the fraction of aggregated cells.

All observations were performed at a temperature of 23 °C with a hematocrit of 10 %. This concentration ensured an almost fully covered bottom surface after 5 min of sedimentation for most sample solutions, with an average coverage of 2.9×10^4 cells/mm^2. Imaging was performed using a confocal microscope (TSC SP5 II; Leica Microsystems, Mannheim, Germany) with a laser of 633nm as the excitation wavelength. RBCs were classified as part of a cluster if the membranes of two cells were optically indistinguishable over a distance of at least 4 pixels, which corresponds to 1 μm in our image with a size of 144 x 144 μm^2. White-light microscopy was used as a control to exclude any possible influence of CellMask on aggregation (data not shown). Fibrinogen and dextran solutions were compared to PBS as negative control. Due to the increase of viscosity with increase in dextran or fibrinogen concentration, the sedimentation slows down, which leads to a lower total number of cells per image at higher viscosities in comparison to RBCs in PBS.

Figure 1(a) shows representative images for various concentrations of dextran and fibrinogen.

In dextran solutions, the number of *rouleaux* and the MAI exhibit a bell shaped curve with a maximum around a concentration of 50 mg/ml and almost no *rouleaux* at 120 mg/ml. (Fig. 1(b)). For fibrinogen, a monotonous increase of the MAI is seen up to a concentration of 12mg/ml, which corresponds to the physiological range. Both results are reminiscent of the dependencies of interaction energy, in-flow clustering and rheology on dextran concentration obtained in previous studies[2,3]. Another qualitative observation is that the dextran and fibrinogen induced *rouleaux* look slightly different with more interconnections in the fibrinogen case.

Contact zone contour extraction

All measurements of contact zone shapes of RBC clusters were performed at 0.2% v/v hematrocrit. The procedure for extracting interfacial shapes for further analysis is exemplified in Fig. 2. The contact zones of RBC clusters are first isolated. The images are then thresholded and skeletonized to extract the interface shape on which a sine function can be fitted to measure the amplitude of deformation.

Echinocyte clusters

When the doublets were allowed to sediment on the BSA treated cover slip, the cells remained in discocyte state for several minutes but they were mostly oriented with their interface parallel to the coverslip which did prevent a characterization of the thin interfacial membrane that was then oriented perpendicular to the z-axis of the point spread function. This was different for the non-treated cover slips: due to the so called "glass effect", the RBCs transformed into the echinocyte state and we observed only male-female and sigmoid shapes (Fig. 3(a)). A quantitative analysis of the distribution of the different interfacial shapes for the different concentrations of dextran is shown in Fig. 3(b). For all different cases we observed an increase in the number of sigmoid interfaces in the intermediate range of polymer concentrations, i.e. at larger interaction strengths. The sigmoid shape allows for the largest contact zone, favorized by large interaction strength. At low and high concentrations of dextran we observe only few sigmoid shapes and more parachute than male-female shapes for the cells in discocytic state. With fibrinogen, mostly parachute shapes were observed. This is most likely related to the low (but physiological) concentrations of the protein.

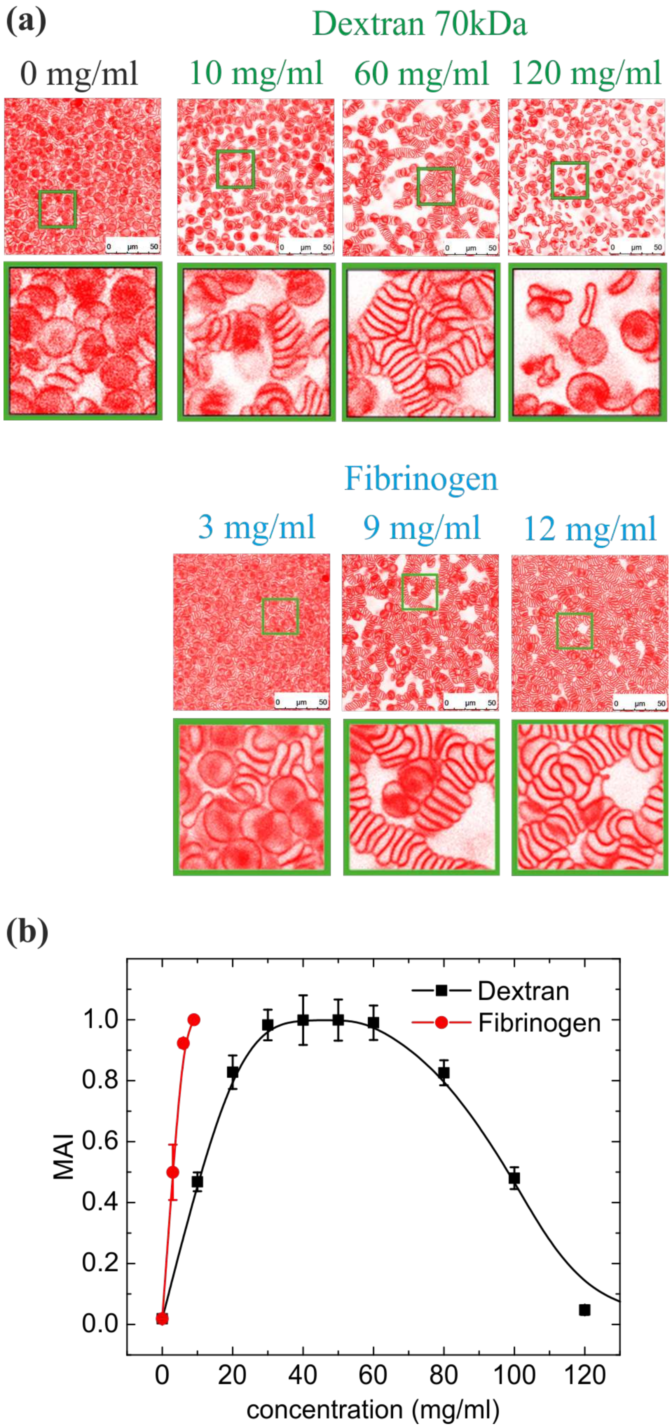

Figure 1. (a) Confocal images of RBCs at 10% hematocrit on the cover slip after 5 minutes of sedimentation at different concentrations of dextran and fibrinogen. The second row of images shows enlargements of the first row indicated by the green frames. (b) The microscopic aggregation index. Error bars represent the standard deviations.

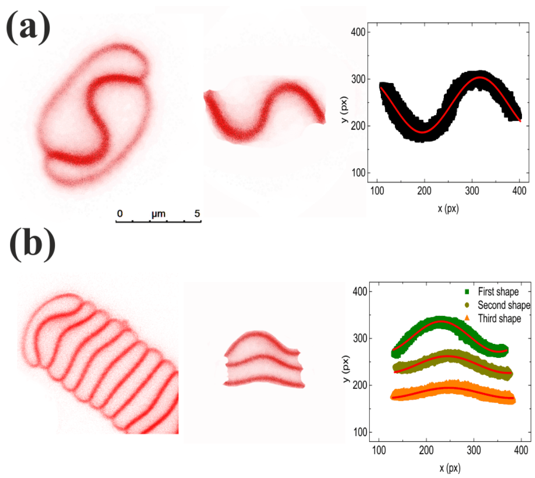

Figure 2. (a) Contact zone shape extraction from doublet clusters (b) Contact zone shape extraction from rouleaux.

For cells in echinocyte state no parachute shape was observes which is to be expected in view of the spherical shape of a single echinocyte.

Modelling and numerical methods

2D numerical model

Based on several successful studies (especially equilibrium shapes and shape dynamics under external flow) that have shown that 2D and 3D models capture the same essential features[4–6], have restricted ourselves here to a 2D model. In 2D the notion of in-plane shear elasticity looses its meaning and the vesicles and inextensible capsule model (often evoked to model RBC) are equivalent. Vesicles consist of closed bilayer membranes enclosing an inner fluid and suspended in an outer fluid. Using the typical reduced area (equivalent of the surface-to-volume ratio in three dimensions) of the RBC, the minimum energy shape of an unstressed vesicle is the same as for an RBC despite the lack of a cytoskeleton. Indeed, the stress free shape is dictated by the curvature energy and the rigidity of the cell. To describe the mechanical behavior of the vesicle, we employ (for each cell) the two dimensional Helfrich free energy[7,8] (per unit length in the 3rd dimension):

$$E_B = \oint \frac{\kappa_B}{2}(c-c_0)^2 dl \tag{1}$$

where c is the mean curvature, dl is the arclength element along the membrane contour, κ_B is the membrane bending rigidity modulus, and c_0 is the spontaneous curvature that can be neglected in case of symmetrical membranes; actually in 2D the spontaneous curvature plays no role. In order to fulfill the condition of membrane inextensibility we supplemented the curvature energy with a term $\oint \zeta dl$, where ζ is a Lagrange multiplier that depends on the position along the membrane . The fluid inside and outside the membrane is described by the Stokes equations (the inertial effect is small and is neglected) with boundary conditions at the membrane and far away from the membrane. Far away from the membrane the flow vanishes (in the absence of any imposed external flow). At the membrane we require velocity continuity, and force balance. In addition, the velocity field along the membrane has to be divergence-free in order to enforce membrane incompressibility. This condition represents an implicit equation that allows one to determine the still unknown Lagrange multiplier ζ; the details of how this is achieved in practice can be found in[9]. The hydrodynamics stress jump across is counterbalanced by the membrane force. This force is composed of the bending force obtained as a functional derivative of the Helfrich free energy supplied with a tension-like force to fulfill the condition of inextensibility of the membrane.

$$\mathbf{f}(\mathbf{X}) = \kappa_B[\frac{\partial^2 c}{\partial l^2} + \frac{c^3}{2}]\mathbf{n} - c\zeta\mathbf{n} + \frac{\partial \zeta}{\partial l}\mathbf{t} \tag{2}$$

where $\mathbf{n}$ and $\mathbf{t}$ are the normal and tangent unit vectors. $\mathbf{X}$ designates the vector position of a given point on a given cell; the curvature as well as the Lagrange multiplier are defined for each cell. In addition to this force we had to consider the force due to cell-cell interaction (due to the presence of proteins or dextran). For this purpose we refer to the work of Neu and Meiselman[10]. Neu and Meiselman have presented a model that takes into account the strong electrostatic repulsion due to the negative charge of RBC membranes, and the weak osmotic forces of attraction due to the depletion effect induced by

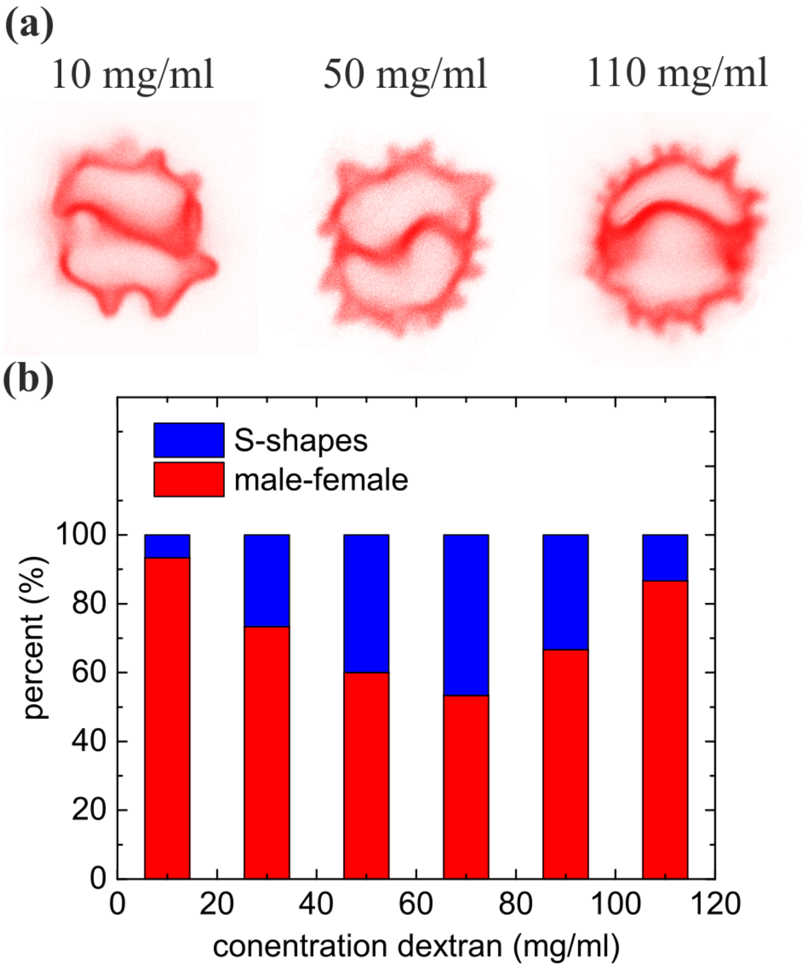

Figure 3. (a) Representative shapes of doublets in echinocytic state, placed on the cover slip without BSA treatment for different concentrations of dextran (from left to right): male-female, S-shape, male-female. (b)Distribution of male-female and S-shapes for echinocyte RBCs in dextran solutions in contact with the cover slip. Parachute shapes were not observed in this case.

macromolecules surrounding RBCs[10]. This model contains several parameters depending on the physiochemical properties and the concentration of the polymers. Often, the interaction energy between cells is described by a Morse potential (or alternatively a Mie type potential), having an exponential form, which has proven to adequately describe experimental data[11]. As is the case in many physical problems, a Morse potential can be well approximated by a Lennard-Jones potential, and we adopted this form in the present study for practical reasons. At a mesoscopic scale (after integration over a distance greater than the cut-off length of the microscopic interaction potential), this reduces to an adhesion energy per unit surface as we shall see below, or as considered in other models (our analytical model and in Ziherl and Svetina[12]) and as can be measured in experiments[2], independent of microscopic details.

The interaction potential per unit (length of the 1st membrane $\times$ length of the 2nd membrane $\times$ length in the 3rd (invariant) dimension) is

$$\phi(r) = 4\varepsilon[(\frac{\sigma}{r})^{12} - (\frac{\sigma}{r})^{6}] \tag{3}$$

where ε is a microscopic parameter with dimension [energy/length^3].

This potential is minimum at distance $r_* = \sigma 2^{1/6}$ and the minimal interaction potential is equal to $\phi_* = -\varepsilon$.

The cell-cell force is given by

$$\mathbf{f}^{\phi}(\mathbf{X}) = -\int_{\sum_{j\neq i}\partial\Omega_j} \frac{\partial\phi(r)}{\partial r}\frac{\mathbf{r}}{r}dl(\mathbf{Y}) \tag{4}$$

$\mathbf{r} = \mathbf{X} - \mathbf{Y}$, $r = \|\mathbf{X} - \mathbf{Y}\|$, and X and Y are two position vectors belonging to the ith and jth membrane ($\partial\Omega$) respectively.

In this formulation, ε is a microscopic parameter of interaction between surface elements. Given the range of the interaction potential (typically a few times σ), an integration of the potential over planar parallel surfaces of extension much larger than this range must be performed in order to derive the macroscopic interaction energy per unit area ε_{ad}.

The force per unit surface between two infinite parallel membranes at distance d is:

$$f(d) = \frac{3\pi\varepsilon}{64}\left(\frac{\sigma}{d}\right)^6\left(160 - 231\left(\frac{\sigma}{d}\right)^6\right) \tag{5}$$

The zero force position is therefore $d_0 = (231/160)^{1/6}\sigma$, which is smaller than r_*. The interaction energy per unit surface between infinite plates (*i.e.* whose extension is much larger than d_0) is then:

$$\varepsilon_{ad} = -\int_{-\infty}^{\infty} \phi\left(\sqrt{x^2 + d_0^2}\right) dx = \frac{48 \times 5^{5/6} \times 6^{1/6}}{11 \times 77^{5/6}} \pi\varepsilon\sigma \simeq 1.89\varepsilon\sigma \tag{6}$$

This gives the relation between the microscopic parameters of the potential ε and σ and the macroscopic interaction energy per unit area ε_{ad}.

Recently the RBC-RBC adhesion energy has been quantified using the atomic force microscopy-based single cell force spectroscopy[2]. From these data, in principle, we can determine the adhesion energy ε_{ad} and equilibrium distance d_0 from which the two parameters entering the Lennard-Jones potential can be determined, namely the minimal energy ε and the range of the potential σ. However, here we chose to adapt the results from the numerical simulations, namely the amplitude of the interfacial deformation, directly to the experimental data and use this to deduce the interaction energy. In this procedure, due to numerical constraints, the zero force length (i.e. the length corresponding to minimal potential) was fixed in our simulations to 489 nm. This was probably significantly larger than the experimental value but still in the order of the optical resolution limit. In addition, note that as long as σ is small compared to the lateral extension of the membrane, the product $\varepsilon\sigma$ is the most quantity here. The Stokes equations were solved using the boundary integral technique as presented in [6,13]

$$\mathbf{u}(\mathbf{X_0}) = \frac{1}{2\pi\mu_1(1+\lambda)} \int_{\sum_i \partial\Omega_i} \mathbf{G}(\mathbf{X},\mathbf{X_0})[\mathbf{f}(\mathbf{X}) + \mathbf{f}^{\phi}(\mathbf{X})]dl(\mathbf{X}) + \frac{(1-\lambda)}{2\pi(1+\lambda)} \int_{\sum_i \partial\Omega_i} \mathbf{u}(\mathbf{X}) \cdot \mathbf{T}(\mathbf{X},\mathbf{X_0}) \cdot \mathbf{n}(\mathbf{X})dl(\mathbf{X}) \tag{7}$$

where $\mathbf{u}(\mathbf{X_0})$ is the velocity of a vector position $\mathbf{X_0}$ belonging to the membrane $\partial\Omega_i$, λ is the viscosity contrast between the inner and outer fluids with the respective viscosities μ_2 and μ_1, and $\mathbf{G}$ and $\mathbf{T}$ are the Green's functions associated with the velocity and the stress fields respectively.

To match the average values of a human RBC, the reduced area (τ) of the vesicle typically lies in the range $(0.60, 0.65)$, the typical radius is $R_0 = 3\mu m$, the membrane stiffness is $\kappa_B = 4 \times 10^{-19} J$. Here we did not investigate any dynamic effects and we therefore fixed the viscosity ratio simply to unity, even if typically a factor of 5 was assumed. We considered doublets and sets of 7 cells, separated by an initial surface-to-surface interdistance of 600 nm.

3D numerical model

The RBC is considered as a two-dimensional hyper-elastic membrane embedded with resistances to area dilatation, volume changes, shear and bending deformations. The membrane is discretized using flat triangles and forces are evaluated on each membrane node. We use 1620 triangles and 812 nodes to discretize each red blood cell.

The bending force is derived from the Helfrich free energy[8] and reads as

$$\mathbf{f}_b = \kappa_b[2H\{2H^2 - 2K\} + 2\Delta_S H]\mathbf{n} \tag{8}$$

where κ_b is the membrane stiffness, H and K are the main and Gaussian curvatures, Δ_S is the Laplace-Beltrami operator and $\mathbf{n}$ is the normal vector pointing outward. We use a discrete differential geometry operators approach proposed by[14] to compute numerically the Helfrich force. Detailed reviews on the accuracy and performance of different numerical methods for estimating the bending force can be found in[15,16]. The membrane strain energy obeys to the Skalak constitutive law[17] given by

$$W_s = \int dA\{\frac{G_s}{4}[I_1^2 + 2I_1 - 2I_2] + \frac{G_s}{4}CI_2^2\} \tag{9}$$

where $I_1 = \lambda_1^2 + \lambda_2^2 - 2$ and $I_2 = \lambda_1^2\lambda_2^2 - 1$ are the strain invariants, λ_1 and λ_2 are the principal stretch ratios, $C > -1/2$ is a constant, G_s and $K_s = [1-2C]G_s$ are the shear elastic and area dilatation modulii. The conservation of the RBC volume corresponding to the impermeability of the membrane condition is fulfilled using a volume penalty function defined as

$$W_v = \frac{\kappa_v}{2}\frac{(V-V_0)^2}{V_0} \tag{10}$$

where κ_v is a volume modulus expressed in unit of energy per volume, V_0 is the reference volume of the stress-free RBC and V is its actual volume. The shear elastic and penalty volume forces are obtained following the principles of virtual work such that

$$\mathbf{f}_\alpha = -\frac{\partial W_\alpha}{\partial \mathbf{X}} \tag{11}$$

where the subscript α can be substituted by v for the volume force or s for the shear elastic force. The details of the numerical implementation of eq. (11) are described in[18]. Consider the nodes $\mathbf{X} \in \partial\Omega_i$ and $\mathbf{Y} \in S_i$ where $\partial\Omega_i$ is the surface of the ith membrane and $S_i \in \partial\Omega_j$ (with $j \neq i$) is the area of membrane j that is within the cut-off distance of the interaction force from node $\mathbf{X}$. The force between one node of a membrane and the other whole membrane is derived from a Lennard-Jones potential and reads:

$$\mathbf{f}_\phi(\mathbf{X}) = 24\int_{S(\mathbf{Y})}\frac{dS}{r^2}\varepsilon[2(\frac{\sigma}{r})^{12} - (\frac{\sigma}{r})^6]\mathbf{r} \tag{12}$$

where $\mathbf{r} = \mathbf{X} - \mathbf{Y}$ and $r = \|\mathbf{X} - \mathbf{Y}\|$. We use in our code the dimensionless adhesion strength $\Gamma \equiv \varepsilon R_0^2/\kappa_b$ instead of ε which is an energy per node of the considered membrane and per unit surface of the other membrane. In what follows the simulation parameters are expressed in lattice units ($l.u.$). The zero force distance is fixed in all our simulations to $\sigma = 1.5$ $l.u.$. The other parameters read as $G_s = 10^{-2}$ $l.u.$, $\kappa_b = 1.5 \times 10^{-3}$ $l.u.$, $\kappa_v = 1$ $l.u.$ and $R_0 = 8$ $l.u.$ We use a cubic domain with dimensions of $80 \times 80 \times 80$. The cells are initialized as biconcave shapes with reduced volume fixed to $\nu = 0.65$ and separated with a mass-to-mass center distance of 6.5 $l.u.$. They are located initially in the middle of the box.

The Navier-Stokes equations are solved using the discretized form of the lattice Boltzmann D3Q19[19–21] with a BGK collision operator[22].

$$f_i(\mathbf{X}' + \mathbf{c}_i\Delta t, t+\Delta t) - f_i(\mathbf{X}', t) = -\frac{1}{\tau}[f_i(\mathbf{X}', t) - f_i^{(eq)}(\mathbf{X}', t)] + \Delta t f_i^{ext}(\mathbf{X}', t) \tag{13}$$

where $i = 1, ..., 19$, $f_i(\mathbf{r}', t)$ are the particle distribution functions, $\mathbf{c}_i$ is the discrete velocity in the ith direction, and τ is the relaxation time. The last term in the RHS of eq. (13) includes the contribution of the total membrane forces on the fluid and has the unit of force per volume. The lattice constant Δx, the timestep Δt, the reference density ρ_0 and the relaxation time are set to unity.

We use the immersed boundary method (IBM)[18,23] to account for the fluid-structure coupling. The bending force comes in the form of a surface force density and needs to be converted to a force before being plugged in the force spreading equation. This is done by multiplying the density nodal force by its equivalent nodal area ΔA_i.

As for 2D simulations, the microscopic interaction parameter ε needs to be converted into a macroscopic adhesion energy per unit surface ε_{ad}. The force per unit surface between two infinite parallel membranes at distance d is:

$$f(d) = \frac{48\pi\varepsilon}{5}\left(\frac{\sigma}{d}\right)^6\left(1 - \frac{10}{11}\left(\frac{\sigma}{d}\right)^6\right) \tag{14}$$

The equilibrium distance (zero force position) is $d_0 = (10/11)^{1/6}\sigma$ and the interaction energy per unit surface between infinite plates is:

$$\varepsilon_{ad} = -\int_0^\infty \phi\left(\sqrt{\rho^2 + d_0^2}\right)2\pi\rho d\rho = \frac{15 \times 11^{2/3} \times 2^{1/3}}{5^{8/3}}\pi\varepsilon\sigma^2 \simeq 3.75\varepsilon\sigma^2 \tag{15}$$

Analytical model

We provide here the details of the analytical model showing buckling of contact zones in symmetric RBC doublets. We suppose that the shape of an RBC in a symmetric doublet (in 2D) is approximated as shown in Fig. 4, with two semi-circular caps of

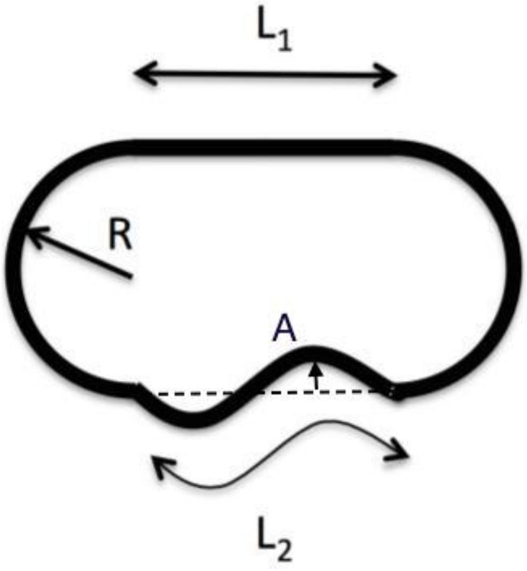

Figure 4. Notations

radius R, a roughly straight portion where curvature is negligible (length L_1) and a contact zone with a sinusoidal shape of curvilinear length L_2 and amplitude A described by the equation:

$$y(x) = AL_1 \sin \frac{2\pi x}{L_1} \tag{16}$$

for $-L_1/2 < x < L_1/2$. The corresponding interaction energy of this contact zone is in expansion at order 4 in A:

$$L_2 = \int_{-L_1/2}^{L_1/2} \left(1 + 2(\pi A)^2 \cos^2 \frac{2\pi x}{L_1}\right) dx = L_1 \left(1 + A^2\pi^2 - \frac{3}{4}\pi^4 A^4\right) \tag{17}$$

This corresponds to an interaction energy E_i:

$$E_i = -\varepsilon_{ad} L_1 \left(1 + A^2\pi^2 - \frac{3}{4}\pi^4 A^4\right). \tag{18}$$

where ε_{ad} is the interaction energy per unit surface.

The curvature energy of the system is concentrated in the circular caps E_{b1} and the deformed contact zone E_{b2}. The circular cap term is:

$$E_{b1} = \frac{\kappa}{R^2} 2\pi R = \frac{2\pi\kappa}{R} \tag{19}$$

The curvature of the contact zone is:

$$C(x) = -\frac{y''(x)}{(1 + (y'(x))^2)^{3/2}}. \tag{20}$$

This leads to the energy term:

$$
\begin{aligned}
E_{b2} &= \kappa \int_{-L_1/2}^{L_1/2} C^2(x)(1+(y'(x))^2)^{1/2} dx \\
&= \kappa \int_{-L_1/2}^{L_1/2} \frac{16\pi^4 A^2 \sin^2\left(\frac{2\pi x}{L_1}\right)}{L_1^2\left(4\pi^2 A^2 \cos^2\left(\frac{2\pi x}{L_1}\right)+1\right)^{5/2}} dx \\
&\simeq \kappa \frac{8\pi^4 A^2 - 20\pi^6 A^4}{L_1}
\end{aligned}
$$

The total energy $E = E_i + E_{b1} + E_{b2}$ therefore depends on A, R and L_1 which are related through the constant area S and constant perimeter L constraints

$$
\begin{aligned}
L &= 2\pi R + L_1(2 + A^2\pi^2 - \frac{3}{4}\pi^4 A^4) && (21)\\
S &= \pi R^2 + 2RL_1 && (22)
\end{aligned}
$$

In the following we define a dimensionless energy $E^* = EL/\kappa$, a rescaled interaction energy $\beta = \varepsilon_{ad} L^2/\kappa$, and rescale all lengths by L: $R* = R/L$ and so on, and use the definition of the reduced area $\tau = 4\pi S/L^2$.

The constant perimeter and area constraints can then be written as the following (dropping the * for simplicity):

$$
\begin{aligned}
1 &= 2\pi R + L_1(2 + A^2\pi^2 - \frac{3}{4}\pi^4 A^4) && (23)\\
\tau/4\pi &= \pi R^2 + 2RL_1 && (24)
\end{aligned}
$$

and can be used to eliminate R and L_1 as in equations 25 and 26.

$$
\begin{aligned}
R &= -\frac{\pi^3 A^4\left(2\tau + \sqrt{1-\tau} - 1\right)}{16\sqrt{1-\tau}} + \frac{1}{4}A^2\left(\pi - \pi\sqrt{1-\tau}\right) + \frac{1-\sqrt{1-\tau}}{2\pi} && (25)\\
L_1 &= \frac{1}{2}\sqrt{1-\tau} - \frac{\pi^2 A^2}{4} + \frac{A^4\pi^4\left(-\tau + 3\sqrt{1-\tau} + 2\right)}{16\sqrt{1-\tau}} && (26)
\end{aligned}
$$

The total energy E^* can then be derived and expanded at order 4 in A as in equation **??**, dropping the * for simplicity.

$$
\begin{aligned}
E = &-\frac{4\pi^2}{\sqrt{1-\tau}-1} - \frac{1}{2}\beta\sqrt{1-\tau} \\
&+ A^2\left(-\frac{1}{2}\pi^2\beta\sqrt{1-\tau} + \frac{\pi^2\beta}{4} + \frac{16\pi^4}{\sqrt{1-\tau}} + \frac{2\pi^4}{\sqrt{1-\tau}-1}\right) \\
&+ A^4\Bigg(-\frac{5\pi^4\beta\tau}{16\sqrt{1-\tau}} + \frac{\pi^4\beta}{4\sqrt{1-\tau}} + \frac{\pi^4\beta}{16} - \frac{7\pi^6\tau}{2\left((\sqrt{1-\tau}-1\right)^3\sqrt{1-\tau}} \\
&\quad + \frac{2\pi^6\tau}{\left(\sqrt{1-\tau}-1\right)^3}\frac{3\pi^6}{\left(\sqrt{1-\tau}-1\right)^3\sqrt{1-\tau}} - \frac{40\pi^6}{\sqrt{1-\tau}} + \frac{8\pi^6}{1-\tau} - \frac{3\pi^6}{\left(\sqrt{1-\tau}-1\right)^3}\Bigg)
\end{aligned}
$$

This energy has only a minimum for a finite, critical value of the rescaled interaction energy β.

If we solve $\partial E/\partial A = 0$ to find the equilibrium configurations we get the two solutions whose expressions are given by 27, which defines the bifurcation threshold β_c corresponding to $A_{eq} = 0$ (Eq. 28)

$$A = \begin{cases} 0 \text{ for } \beta < \beta_c \\ \pm \dfrac{\sqrt{\beta\left(4\sqrt{1-\tau}-2\right)+16\pi^2\left(\frac{1}{1-\sqrt{1-\tau}}-\frac{8}{\sqrt{1-\tau}}\right)}}{\pi\sqrt{\frac{\beta\tau\left(-5\sqrt{1-\tau}\tau-\tau+4\sqrt{1-\tau}+1\right)+8\pi^2\left(5\left(\sqrt{1-\tau}+1\right)+\left(11-84\sqrt{1-\tau}\right)\tau\right)}{(1-\tau)\tau}}} \text{ for } \beta \geq \beta_c \end{cases} \tag{27}$$

$$\beta_c = \frac{8\pi^2\left(9\sqrt{1-\tau}-8\right)}{-2\sqrt{1-\tau}\tau+3\tau+3\sqrt{1-\tau}-3} \tag{28}$$

References

1. Jan, K.-M. & Chien, S. Role of surface electric charge in red blood cell interactions. *J. Gen. Physiol.* **61**, 638–654 (1973).
2. Steffen, P., Verdier, C. & Wagner, C. Quantification of depletion-induced adhesion of red blood cells. *Phys. Rev. Lett.* **110**, 018102 (2013).
3. Brust, M. *et al.* The plasma protein fibrinogen stabilizes clusters of red blood cells in microcapillary flows. *Sci. Rep.* **4**, 4348 (2014).
4. Kaoui, B., Biros, G. & Misbah, C. Why do red blood cells have asymmetric shapes even in a symmetric flow? *Phys. Rev. Lett.* **103**, 188101 (2009).
5. Fedosov, D. A., Peltomaki, M. & Gompper, G. Deformation and dynamics of red blood cells in flow through cylindrical microchannels. *Soft Matter* **10**, 4258–4267 (2014).
6. Aouane, O., Thiébaud, M., Benyoussef, A., Wagner, C. & Misbah, C. Vesicle dynamics in a confined poiseuille flow: From steady state to chaos. *Phys. Rev. E* **90**, 033011 (2014).
7. Canham, P. The minimum energy of bending as a possible explanation of the biconcave shape of the human red blood cell. *J. Theor. Biol.* **26**, 61–81 (1970).
8. Helfrich, W. Elastic properties of lipid bilayers: theory and possible experiments. *Z. Naturforsch. C* **28**, 693–703 (1973).
9. Ghigliotti, G., Biben, T. & Misbah, C. Rheology of a dilute two-dimensional suspension of vesicles. *J. Fluid Mech.* **653**, 489–518 (2009).
10. Neu, B. & Meiselman, H. Depletion-mediated red blood cell aggregation in polymer solutions. *Biophys. J.* **83**, 2482–2490 (2002).
11. Liu, Y. & Liu, W. K. Rheology of red blood cell aggregation by computer simulation. *J. of Comput. Phys.* **220**, 139–154 (2006).
12. Ziherl, P. & Svetina, S. Flat and sigmoidally curved contact zones in vesicle–vesicle adhesion. *Proc. Natl. Acad. Sci. USA* **104**, 761–765 (2007).
13. Thiébaud, M. & Misbah, C. Rheology of a vesicle suspension with finite concentration: A numerical study. *Phys. Rev. E* **88**, 062707 (2013).
14. Meyer, M., Desbrun, M., Schröder, P., Barr, A. H. *et al.* Discrete differential-geometry operators for triangulated 2-manifolds. *Visualization and mathematics* **3**, 52–58 (2002).
15. Tsubota, K.-i. Short note on the bending models for a membrane in capsule mechanics: comparison between continuum and discrete models. *Journal of Computational Physics* **277**, 320–328 (2014).
16. Guckenberger, A., Schraml, M. P., Chen, P. G., Leonetti, M. & Gekle, S. On the bending algorithms for soft objects in flows. *Computer Physics Communications* **207**, 1–23 (2016).
17. Skalak, R., Tozeren, A., Zarda, R. & Chien, S. Strain energy function of red blood cell membranes. *Biophysical Journal* **13**, 245–264 (1973).
18. Krüger, T., Varnik, F. & Raabe, D. Efficient and accurate simulations of deformable particles immersed in a fluid using a combined immersed boundary lattice boltzmann finite element method. *Computers & Mathematics with Applications* **61**, 3485–3505 (2011).

19. Succi, S. *The lattice Boltzmann equation: for fluid dynamics and beyond* (Oxford university press, 2001).

20. Harting, J., Chin, J., Venturoli, M. & Coveney, P. V. Large-scale lattice boltzmann simulations of complex fluids: advances through the advent of computational grids. *Philosophical Transactions of the Royal Society of London A: Mathematical, Physical and Engineering Sciences* **363**, 1895–1915 (2005).

21. Aidun, C. K. & Clausen, J. R. Lattice-boltzmann method for complex flows. *Annual review of fluid mechanics* **42**, 439–472 (2010).

22. Bhatnagar, P. L., Gross, E. P. & Krook, M. A model for collision processes in gases. i. small amplitude processes in charged and neutral one-component systems. *Physical review* **94**, 511 (1954).

23. Peskin, C. S. The immersed boundary method. *Acta numerica* **11**, 479–517 (2002).
